# Integrating Immersive Virtual Reality With Savoring to Promote the Well-Being of Patients With Chronic Respiratory Diseases: Pilot Randomized Controlled Trial

**DOI:** 10.2196/67395

**Published:** 2025-09-23

**Authors:** Elisa Pancini, Alessia Fumagalli, Sveva Maggiolini, Clementina Misuraca, Davide Negri, Luca Bernardelli, Daniela Villani

**Affiliations:** 1 Research Center on Communication Psychology, Department of Psychology, Università Cattolica di Milano, Largo Gemelli 1, Milano, 20123, Italy, 39 0272342284; 2 Pulmonary Rehabilitation Unit of IRCCS INRCA, Casatenovo, Italy; 3 School of Medicine and Surgery, University of Milano Bicocca, Milan, Italy; 4 UOC Pneumologia, Fondazione IRCCS San Gerardo dei Tintori, Monza, Italy; 5 Become-Hub, Milan, Italy; 6 Research Unit in Digital Media, Psychology and Well-Being, Department of Psychology, Università Cattolica di Milano, Milan, Italy

**Keywords:** chronic respiratory diseases, psychological well-being, virtual reality, savoring, positive emotions, relaxation, randomized controlled trial, effectiveness, interventions, pulmonary rehabilitation, VR acceptance

## Abstract

**Background:**

Chronic respiratory diseases (CRDs) are widespread pathologies that cause nonreversible airflow limitations as well as extrapulmonary adverse effects. These pathologies are related to frequent hospitalizations and consequently high levels of anxiety, depression, and stress. In this respect, immersive virtual reality (IVR) relaxation integrated with savoring, which is the ability to generate and amplify positive emotions, can enhance well-being and relaxation in patients with CRDs.

**Objective:**

This pilot randomized controlled trial aimed to investigate the effectiveness of a 2-week IVR-based relaxation intervention integrated with savoring in patients with CRDs for increasing emotional and psychological well-being, positive emotions, relaxation, and peripheral oxygen saturation (SpO_2_), and decreasing negative emotions.

**Methods:**

This study included 45 hospitalized patients with CRDs from the Pulmonary Rehabilitation Unit of Istituto di Ricovero e Cura a Carattere Scientifico (IRCCS) Istituto Nazionale di Ricovero e Cura per Anziani (INRCA) Casatenovo. Alongside traditional pulmonary rehabilitation, the experimental group (n=23) took part in a 4-session IVR-based intervention, while the active control group (n=22) listened to relaxing music. In each session, the experimental group experienced a relaxing virtual scenario followed by a savoring exercise. Both groups completed self-reported questionnaires at 3 time points—preintervention/baseline (T0), postintervention (T1), and 1-month follow-up (T2)—as well as before and after each session. The experimental group’s IVR acceptance and sense of presence were also measured.

**Results:**

Regarding the primary outcomes, taking T0 and T1 into account, repeated measures analysis of covariance revealed significant increases for the experimental group in emotional well-being (*P*<.001; partial *η*²=0.398), psychological well-being (*P*<.001; partial *η*²=0.559), positive emotions (*P*<.001; partial *η*²=0.407), and relaxation (*P*<.001; partial *η*²=0.598), and a significant decrease in negative emotions (*P*<.001; partial *η*²=0.456) compared to the control group. Moreover, 2-tailed paired *t* tests, considering T0 and T2, revealed significant long-term psychological changes at T2 for the experimental group in emotional well-being (*P*=.046), psychological well-being (*P*=.03), and positive emotions (*P*=.005), whereas the control group reported no significant changes. Concerning secondary outcomes, no significant differences in SpO_2_ between the 2 groups were found, and patients in the experimental group reported high IVR acceptance and sense of presence.

**Conclusions:**

These results suggest that relaxing IVR integrated with savoring may promote well-being not only after the intervention but also in the long term. Savoring may have played a role in enhancing the positive effects of the IVR experience by helping patients focus on and amplify positive experiences, thereby mitigating the negative psychological impact of CRDs. However, the study design (control group was exposed only to relaxing music without a savoring component) precludes the ability to determine the specific contribution of each element (IVR and savoring). Future research should aim to disentangle these components in order to clarify their individual and combined effects.

## Introduction

### Background

Chronic respiratory diseases (CRDs) are conditions that negatively impact the lungs and airways [1]. They include chronic obstructive pulmonary disease (COPD), interstitial lung diseases, bronchiectasis, idiopathic pulmonary fibrosis, and chronic asthma [2,3]. CRDs were the third greatest cause of mortality in 2019, accounting for 4.0 million deaths and a global prevalence of 454.6 million cases [4]. These disorders result in frequent and chronic respiratory inflammations and infections [5] and can be caused by continuous exposure to environmental stimuli such as smoke from cigarettes, air pollution, and job-related risks [2]. People with CRDs often experience symptoms, such as cough, dyspnea, breathlessness, and decline in lung function, which limit functional capacity, reduce exercise tolerance, and lead to frequent hospitalizations [2,6,7]. As a result, several patients with CRDs can experience restrictions in their daily activities due to the need to receive daily home therapies such as oxygen therapy [8]. To improve the physiological conditions and physical activities of people with CRDs, pulmonary rehabilitation can be delivered through exercise training accompanied by education and behavior change [2,9]. Moreover, pulmonary rehabilitation can be delivered in an inpatient setting [10] by providing daily exercise training during cyclical hospitalizations.

Because of all these factors, the quality of life of patients with CRDs is adversely affected, and patients frequently report high levels of anxiety and stress, as well as high levels of depression and low levels of emotional and psychological well-being [11,12]. Anxiety and stress, in particular, can have severe impacts on patients’ quality of life and functional capacity [13,14], thus increasing dyspnea and impairing compliance with home therapies [11]. In this context, relaxation promotion can be an effective way to decrease anxiety and stress levels in patients with CRDs. In fact, several studies have shown that listening to relaxing music is effective in relieving anxiety, stress, and dyspnea; improving lung capacity; and promoting greater exercise tolerance [15-18].

### Promoting Relaxation With Virtual Reality

Another promising tool to promote relaxation in patients with CRDs is immersive virtual reality (IVR). This technology enables the creation of interactive worlds generated by computers that replace real-world sensory experiences with digitally created ones, generating the sensation of being in digital settings [19]. IVR involves several key processes, including the sense of presence, which is the feeling of being there in the digital experience [20-22]. Virtual reality (VR) has been successfully employed in promoting relaxation and reducing stress, anxiety [20,23,24], and depression [25-27]. It has also shown relevant potential in improving and regulating emotional well-being and mood, as well as promoting positive emotions [28-31] in patient populations [32,33]. Owing to IVR, users can immerse themselves in various digital environments, such as restorative natural scenarios, and this can be particularly suitable for hospitalized patients who cannot move to natural outside environments [30]. Specifically, virtual restorative scenarios usually show pleasant and peaceful natural environments, such as islands, the sea, parks, and gardens. They can be integrated with nature-related auditory elements with relaxation properties, and they have been shown to be effective in different contexts [29]. To date, however, for patients with CRDs, IVR has been mainly used to facilitate pulmonary rehabilitation, as immersive scenarios enable “attention shifting,” distract patients from negative feelings (eg, fatigue, dyspnea, and monotony), and motivate them during exercise training [34-36]. To our knowledge, only a recent randomized controlled trial (RCT) evaluated the effectiveness of a 2-week, 10-session IVR therapy for the symptoms of depression, anxiety, and stress in hospitalized patients with COPD [7]. A virtual garden based on the Ericksonian psychotherapy approach, representing symbolically the patient’s health and its improvement during hospitalization through changes in the coloring of the scenario, was used. By comparing this VR intervention with a control intervention involving 10 sessions of Schultz autogenic training, the analysis showed significant changes in depression, anxiety, and stress levels in the experimental group.

### Amplification of Positive Emotions

To enhance the positive effect of IVR, an additional tool related to the amplification of positive emotions can be used [24]. Indeed, positive emotions can play an important role in counteracting the negative effects of anxiety and stress in patients with CRDs. In this respect, according to the broaden and build theory of positive emotions, positive emotions broaden habitual ways of thinking and acting [37]. Furthermore, according to the undoing hypothesis, positive emotions can neutralize the harmful effects of negative emotions, and as a consequence, they allow the increase of intellectual resources and build a pool of resources from which to draw in future difficult situations [37-39]. A positive psychology technique strictly related to this approach is savoring, which refers to the ability to recognize, appreciate, and amplify positive experiences and emotions in one’s life [40]. Interestingly, people can savor a positive past event, relive in the present the same positive emotions experienced in the past, and experience new positive emotions (positive reminiscence) [40]. Moreover, they can savor the present moment or a future positive experience (positive anticipation). Literature has shown that savoring interventions consistently increase positive emotions and well-being, decrease negative emotions, and reduce depression [40-44]. Up to now, savoring has not been applied to patients with CRDs, and its possible integration with VR could promote patients’ awareness of the positive states experienced during the virtual experience and enable them to create a connection with their personal experiences.

### Objectives and Hypothesis

Considering these factors and taking advantage of the potential of VR and savoring, we conducted a pilot RCT aimed at investigating the effectiveness of an innovative 2-week intervention involving IVR-based relaxation integrated with savoring in patients with CRDs. This RCT was conducted in collaboration with the Italian VR company Become-Hub that provided the software and the IVR equipment, and the Pulmonary Rehabilitation Unit of the Istituto di Ricovero e Cura a Carattere Scientifico (IRCCS) Istituto Nazionale di Ricovero e Cura per Anziani (INRCA) Casatenovo. Specifically, taking the primary outcomes into consideration from preintervention/baseline (T0) to postintervention (T1), we hypothesized that emotional and psychological well-being would increase more in the experimental group compared to the active control group that listened to relaxing music (hypothesis 1). In addition, we hypothesized that patients in the experimental group would achieve higher levels of positive affect and lower levels of negative affect (hypothesis 2), and higher levels of relaxation (hypothesis 3) compared to the active control group. We also hypothesized that the experimental group would achieve better improvements in psychological changes at the 1-month follow-up (T2) from T0 compared to the active control group (hypothesis 4).

As secondary outcomes, taking pre- and postsessions into consideration, we hypothesized that self-reported relaxation and peripheral oxygen saturation (SpO_2_) would increase more in the experimental group compared to the active control group (hypothesis 5). Through self-report, we also evaluated VR acceptance, the sense of presence experienced in IVR, and the perceived usefulness of the intervention. Finally, we qualitatively explored patients’ experiences with IVR and the savoring exercises.

## Methods

### Ethical Considerations

This study was conducted in compliance with relevant guidelines and regulations in the Declaration of Helsinki [45] and approved by the Ethics Committee of IRCCS INRCA Ancona (protocol number: 3739_2023). Due to administrative issues, trial registration was not completed prospectively. The study was retrospectively registered in the Australian New Zealand Clinical Trials Registry (ACTRN12624000435583) before data analysis and manuscript writing. All participants signed an informed consent form before the start of the study, and data protection and privacy were maintained according to the General Data Protection Regulation (GDPR; EU 2016/679). To ensure confidentiality, data were anonymized and deidentified prior to analysis, and no personally identifiable information was disclosed. The study’s objectives, confidentiality, and anonymity and the data handling procedure were described, and participants were given full authority to complete the study. No financial or other compensation was provided for participation.

### Study Design and Participants

This RCT used a between-subjects design. It included 3 assessment points (ie, T0, T1, and T2) and was carried out from January 2023 to September 2023. G*Power 3.1.9.7 software [46] was used to calculate the sample size. The calculation was based on a repeated-measures ANOVA focusing on the within-between interaction. The type I error rate was set at 5% (*α*=.05), with a medium effect size (*f*=0.25) for the primary outcomes. To achieve a power of 90% with 2 groups and 2 repeated sets of measurements, a correlation of 0.5 among repeated measures and a nonsphericity correction factor ɛ of 1.0 were assumed [7]. Based on these parameters, a minimum of 46 participants was required. The anticipated attrition rate was estimated to be around 15% [47], and thus, 54 participants were considered for inclusion. Consequently, 54 patients from the Pulmonary Rehabilitation Unit of IRCCS INRCA Casatenovo who met the inclusion and exclusion criteria were recruited by doctors together with the researchers. The inclusion and exclusion criteria are provided in Textbox 1.

The patients were hospitalized for about 3 weeks to undergo pulmonary rehabilitation and were not allowed to leave the ward (directives issued by the hospital as a result of the COVID-19 pandemic). Two patients refused to participate due to personal reasons; thus, 52 patients were randomly assigned by the researchers to the experimental and control groups, using block randomization. A label of A or B (A=intervention, B=control) was assigned to each group (block size=4). Free online software (Research Randomizer 4.0) was used to generate the randomization list. The data were screened for outliers, and none were found. Three patients from the experimental group and 2 patients from the active control group were excluded from the analysis because they did not complete the T1 questionnaires due to early hospital discharge. Moreover, 2 patients from the active control group were excluded from the analysis because of severe physical issues during the hospitalization that greatly impacted their psychological health, necessitating psychotherapeutic consultation. Consequently, there were 23 patients in the experimental group (mean age 71.70, SD 7.14 years) and 22 patients in the control group (mean age 68.18, SD 8.66 years). At baseline, demographic data, such as age, gender, educational level, employment status, marital status, and CRD type, were collected.

Textbox 1. **Inclusion and exclusion criteria.**

**Inclusion criteria**

1. Age older than 18 years
2. A diagnosis of chronic respiratory disease

**Exclusion criteria**

1. Pre-existing medical disorders that would prevent the use of the Oculus Quest 2 headset, such as:
  - the existence of binocular vision anomalies
  - a history of seizures or epileptic conditions
  - the presence of cardiac pacemakers, defibrillators, or implanted devices
  - the use of hearing aids
2. A diagnosis of dementia or cognitive impairment documented in the medical records
3. A Mini-Mental State Examination score of <26

### Measures

To assess cognitive impairment prior to the intervention, the Italian version of the Mini-Mental State Examination (MMSE), consisting of 11 items, was used to evaluate patients’ cognitive functions [48-50]. The maximum score achievable was 30, and the cutoff was set at 26 [51-53]. No patients were excluded.

To assess psychological changes from T0 to both T1 and T2, both groups completed several questionnaires.

### Primary Outcomes

Emotional well-being (defined as enjoyment and absence of unpleasant feelings) and psychological well-being (defined as taking steps toward one’s goals and having a life purpose) [54] were measured using the emotional well-being subscale (EWB; 3 items; *ω*=0.76; *α*=.65) and psychological well-being subscale (PWB; 6 items; *ω*=0.80; *α*=.79), respectively, of the Italian Mental Health Continuum Short Form (MHC-SF) [55,56]. A 6-point Likert scale was used to respond (0 [*never*] to 5 [*every day*]).

Positive and negative affect were measured using the Italian version of the Scale of Positive and Negative Experiences (SPANE) [57,58]. This self-report scale consists of 12 items and is divided into 2 subscales: positive affect (SPANE-P; *ω*=0.83; *α*=.83) and negative affect (SPANE-N; *ω*=0.85; *α*=.84). For all items, a 5-point Likert scale was used to respond (1 [*very rarely or never*] to 5 [*very often or always*]).

Furthermore, changes in relaxation within each session were assessed both subjectively through a 10-point visual analog scale (VAS; relaxation VAS; assessed at T0, T1, and T2, and before and after each session) [59] and objectively through the NONIN Palm Saturimeter that measures SpO_2_ (the proportion of oxygen-saturated hemoglobin to total hemoglobin in the blood; assessed before and after each session).

### Secondary Outcomes

To assess IVR acceptance and the sense of presence experienced in IVR by the experimental group, 2 questionnaires were proposed after each session. The Slater Usoh Steed Presence Questionnaire (SUS) [60] was used to examine the patients’ sense of presence in the virtual environment (*ω*=0.84; *α*=.82). This questionnaire consists of 6 items that can be answered via a 7-point Likert scale (1 [*not at all*] to 7 [*very much*]).

IVR acceptance was also assessed through an ad hoc questionnaire based on the Unified Theory of Acceptance and Use of Technology (UTAUT) model [61] and by measuring behavioral intention (*ω*=0.88; *α*=.88), performance expectancy (*ω*=0.94; *α*=.93), effort expectancy (*ω*=0.95; *α*=.94), and anxiety (*ω*=0.94; *α*=.86). The response scale for all the 10 items was a 7-point Likert scale (1 [*strongly disagree*] to 7 [*strongly agree*]). At the end of each session, patients in the experimental group were asked to freely share their impressions about the virtual experience and to describe the positive events they thought about during the savoring exercise.

Participants rated the intensity of 5 emotions (love, awe, enjoyment, gratitude, and hope) on a 10-point VAS ranging from 1 (*not at all*) to 10 (*very much*) after each session.

A final item was used to assess the perceived usefulness of the intervention (“Referring to the entire intervention, how much do you think it was useful for you?”) on a 5-point Likert scale (1 [*not useful at all*] to 5 [*completely useful*]). Finally, patients were invited to share their opinions and suggestions about the intervention.

### Procedure

After providing informed consent, patients in both groups completed the preintervention questionnaires (T0: MMSE, MHC-SF, SPANE, and relaxation VAS). Then, together with traditional pulmonary rehabilitation, the experimental group (n=23) participated in the IVR-based relaxation and savoring intervention, while the active control group (n=22) listened to relaxing music on their headphones, as in previous studies with this population [7]. The music was selected from previous studies [62,63] and a study involving patients with CRDs [15]. The songs used can be found in Section S1 in Multimedia Appendix 1.

**Figure 1.**
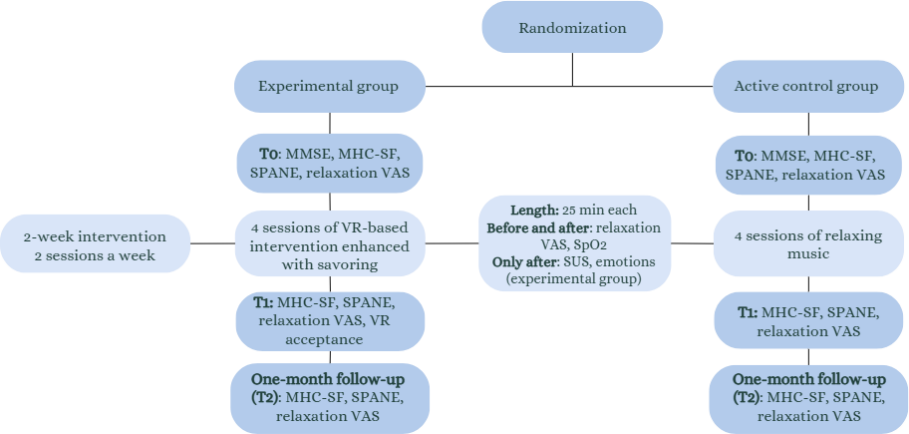
Study procedure. MHC-SF: Mental Health Continuum Short Form; MMSE: Mini-Mental State Examination; SPANE: Scale of Positive and Negative Experiences; SpO_2_: peripheral oxygen saturation; SUS: Slater Usoh Steed Presence Questionnaire; T0: preintervention/baseline; T1: postintervention; VAS: visual analog scale; VR: virtual reality.

The intervention lasted 2 weeks and included four 25-minute sessions in the afternoon after the pulmonary rehabilitation physical exercises (Figure 1). The baseline assessment was performed in a separate session earlier to avoid overloading the patients. In each session, the experimental group experienced an immersive 10-minute relaxing virtual scenario (provided by the Italian VR company Become-Hub) using the Oculus Quest 2 headset integrated with a narrative voice. It was a prerecorded virtual scenario, and patients could explore the virtual environment seated and without using the hand controllers. These scenarios have already been used in other relaxation protocols [26,31,64-66]. In addition, these scenarios have recently received medical CE certification (*Conformité Européenne*, a medical device that meets all applicable health and safety regulations in the European Union).

Moreover, the patients completed a savoring exercise proposed through prerecorded audio after each virtual scenario to consolidate and amplify the positive emotions generated by the IVR experience. The sessions were administered by the researchers and occurred one-on-one with each participant. Furthermore, 4 patients in the experimental group and 3 patients in the control group did not complete the 4th session due to early hospital discharge. At T1 and T2, both groups completed the questionnaires again (MHC-SF, SPANE, and relaxation VAS). At the 1-month follow-up, depending on patients’ preferences, they were contacted by phone to fill out the questionnaires, while those who wanted to complete the questionnaires online could fill them out on Qualtrics.

### IVR and Savoring Protocol

**Figure 2.**
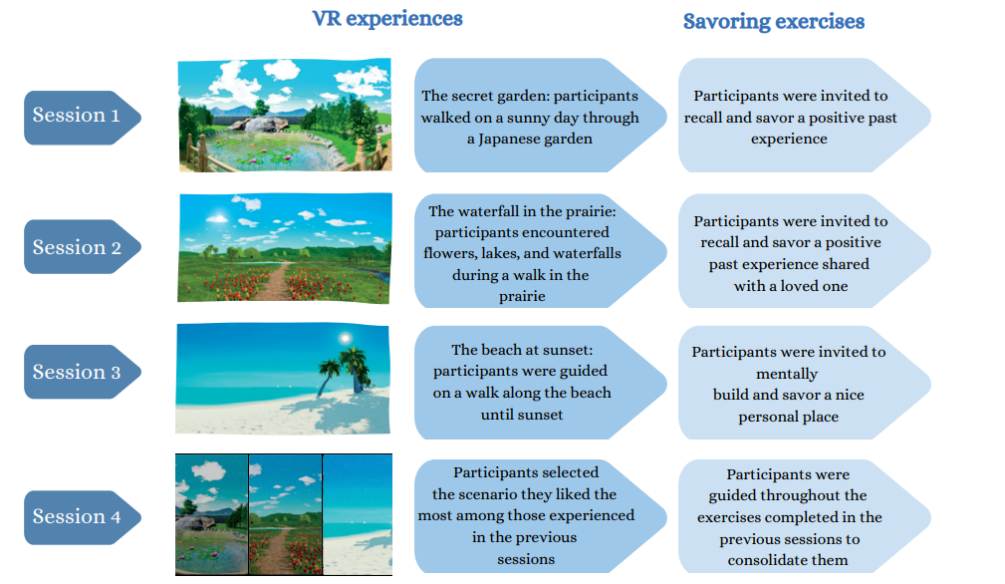
Sessions of the intervention involving immersive virtual reality–based relaxation integrated with savoring. VR: virtual reality.

The protocol included 4 sessions involving IVR-based relaxation integrated with savoring. Specifically, in the first session, the patients watched “the secret garden,” where they were guided through a quiet and peaceful environment. Throughout the virtual scenario, a relaxing narrative voice guided each participant on a walk inside a Japanese garden on a sunny day. Moving forward, it was also possible to cross a calm pond thanks to a small wooden bridge and pass through cherry blossom trees [26,64-66]. After that, the patients were guided through a savoring exercise (an example of the savoring exercise script can be found in Section S2 in Multimedia Appendix 1). In particular, they were invited to remember the positive emotions and sensations felt during the IVR experience. Afterwards, they were asked to recall and savor a positive memory where they felt similar emotions (Figure 2).

In the second session, the patients watched “the waterfall in the prairie” [31]. A relaxing narrative voice made them notice the colorful flowers, the quiet lakes, and the waterfalls during a walk in the prairie. In this environment, they could also listen to the sounds of water, the chirping of birds, and the quiet voices in the distance from a small, distant village. Later, they were invited to recall the positive emotions felt during the virtual experience and were asked to savor a positive past event shared with a loved one.

In the third session, the patients watched “the beach at sunset,” where a narrative voice guided them on a walk along the beach until sunset. In this scenario, they could see the colors of the sand and the sea; the relaxing, soothing hues of the sunset; and the beauty of the starry sky. They could also pay attention to the sound of the waves on the shore and notice the light wind moving the palms. Afterwards, the patients were invited to recall the positive emotions felt during the VR experience and to mentally create and savor a personal nice place connected to these emotions [67,68].

In the fourth session, the patients selected the scenario they liked the most among those experienced in the previous sessions. After that, they were guided through the exercises completed in the previous sessions to consolidate them.

### Data Analysis

Data analysis was conducted using IBM SPSS Statistics (29.0.1.0), and the data were screened for outliers and missing information. Group differences regarding emotional and psychological well-being, positive emotions, negative emotions, and relaxation were examined at baseline using the Student *t* test (2-tailed) for independent samples. The primary outcomes for the experimental group (n=23) and the active control group (n=22) were investigated by considering MHC-SF EWB, MHC-SF PWB, SPANE-P, SPANE-N, and relaxation VAS scores at T0 and T1. A repeated measures analysis of covariance (ANCOVA) was used with group condition (experimental group and active control group) as the between-subjects factor and time (T0 and T1) as the within-subjects factor to investigate the time×group interactions as they would indicate a significant change due to the intervention. To assess the sustained psychological improvements from T0, separate within-subjects Student paired *t* tests were conducted at T2 on a subsample of participants in the experimental group (n=17) and active control group (n=15), who completed the T2 questionnaires. MHC-SF EWB, MHC-SF PWB, SPANE-P, SPANE-N, and relaxation VAS scores at T0 and T2 were considered.

Furthermore, group differences regarding relaxation and SpO_2_ in each of the 4 sessions were investigated by identifying the delta scores (by subtracting the presession scores from the postsession scores) and then using the Student *t* test for independent samples.

Thematic analysis was used to assess qualitative data [69]. To address the research question, a thematic analysis detects, analyzes, and provides patterns of meaning (themes) across the dataset. This method allowed for the concise analysis of a large amount of textual material using a deductive approach in which unexpected themes could emerge “bottom-up” from the data. Relevant data points were found methodically before being classified into prospective themes that were refined, defined, and labeled by 2 judges. Finally, the themes were subjected to a narrative analysis, and they are reported in Multimedia Appendix 1.

## Results

### Study Flow and Sample Characteristics

**Figure 3.**
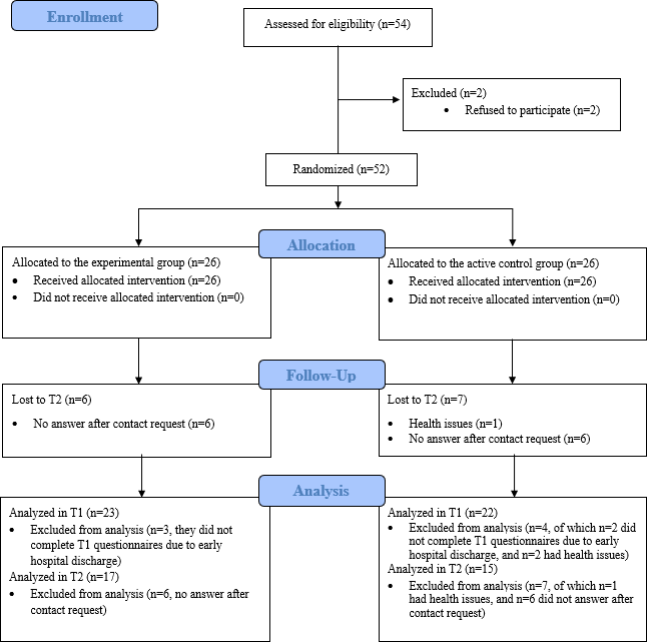
CONSORT (Consolidated Standards of Reporting Trials) flow diagram. T0: preintervention/baseline; T1: postintervention; T2: 1-month follow-up.

The CONSORT (Consolidated Standards of Reporting Trials) flow diagram is presented in Figure 3. The CONSORT checklist is provided in Checklist 1. The characteristics of the final sample (after screening for outliers and missing data) are shown in Table 1.

To investigate differences between groups regarding the psychological dimensions at baseline, the Student *t* test for independent samples was used. Significant differences in emotional well-being (MHC-SF EWB; *t*_43_=–2.27; *P*=.03), psychological well-being (MHC-SF PWB; *t*_43_=–2.62; *P*=.01), and positive emotions (*t*_43_=–4.51; *P*<.001) were found. No significant differences between groups in negative emotions (SPANE-N) and relaxation (relaxation VAS) were found.

**Table 1.** Participant characteristics in the final sample.

Characteristic		Experimental group (N=23)	Control group (N=22)
Age (years), mean (SD)		71.70 (7.14)	69.18 (8.66)
Gender, n (%)			
Male		11 (48)	11 (50)
Female		12 (52)	11 (50)
Education level, n (%)			
Elementary school		4 (17)	6 (27)
Middle school		11 (48)	9 (41)
Senior high school		8 (35)	4 (18)
Bachelor’s degree		—^a^	2 (9)
Master’s degree		—	1 (5)
Marital status, n (%)			
Single		2 (9)	1 (5)
Cohabitant		1 (4)	1 (5)
Married		13 (56)	15 (67)
Divorced		2 (9)	1 (5)
Widowed		5 (22)	4 (18)
Employment status, n (%)			
Worker		1 (4)	1 (5)
Unemployed		—	1 (5)
Retired		20 (87)	19 (85)
Other		2 (9)	1 (5)
Smoking status, n (%)			
Nonsmoker		4 (17)	9 (41)
Active smoker		3 (13)	4 (18)
Ex-smoker		15 (66)	9 (41)
Other		1 (4)	—
Respiratory disease, n (%)			
COPD^b^ mild (FEV1^c^ ≥80%)		2 (9)	—
COPD moderate (79%> FEV1 ≥50%)		4 (17)	5 (23)
COPD severe (49%> FEV1 ≥30%)		7 (30)	7 (31)
COPD very severe (FEV1 <29%)		2 (9)	—
Idiopathic pulmonary fibrosis		3 (13)	—
Chronic asthma		2 (9)	1 (5)
Bronchiectasis		1 (4)	3 (14)
Interstitial lung disease		—	2 (9)
Other		2 (9)	4 (18)

a Not applicable.

b COPD: chronic obstructive pulmonary disease.

c FEV1: forced expiratory volume in 1 second.

### Effectiveness of the Intervention Involving VR-Based Relaxation Integrated With Savoring

**Figure 4.**
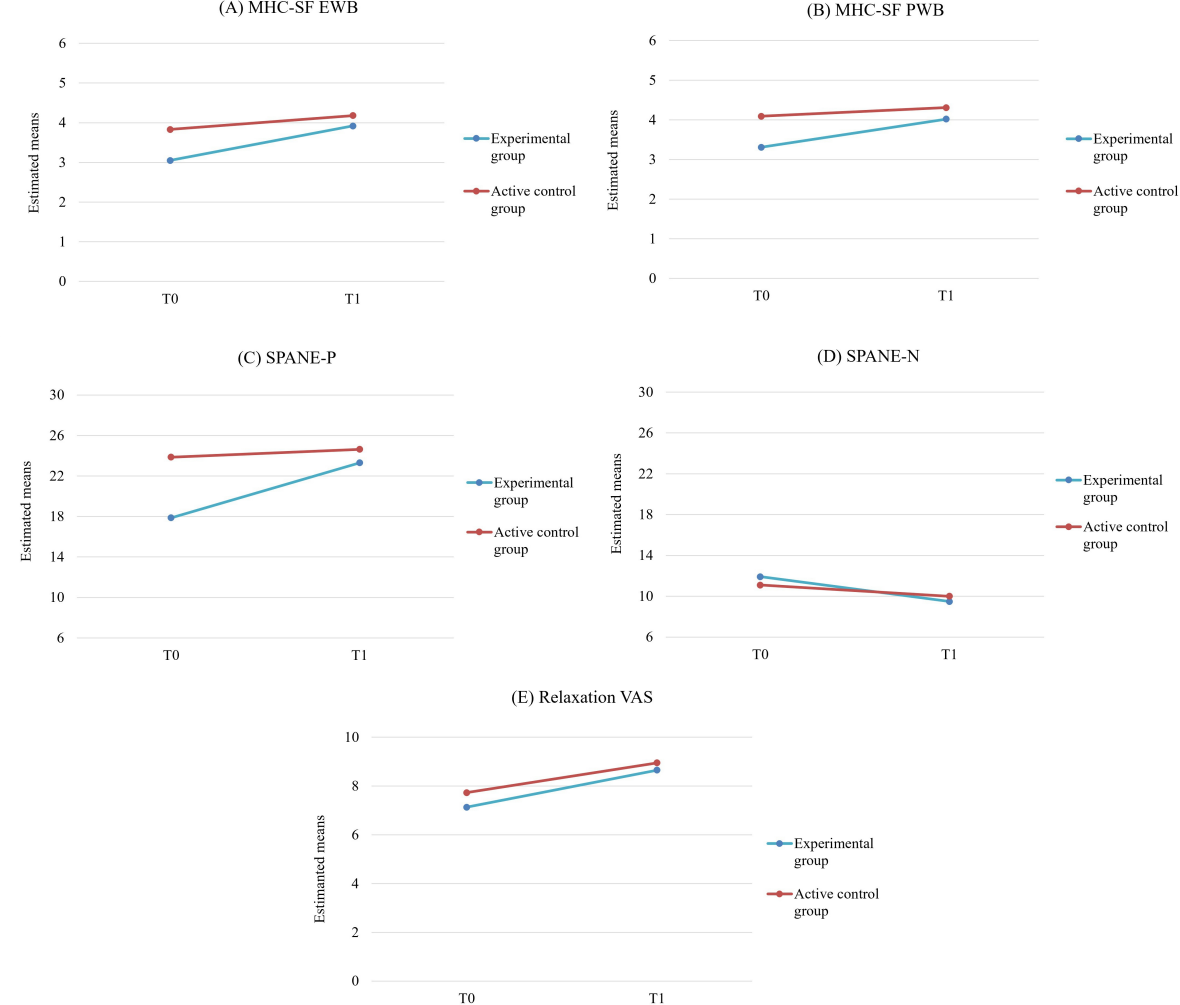
Scores of emotional (A) and psychological (B) well-being, positive (C) and negative (D) emotions, and relaxation (E) at T0 (preintervention/baseline) and T1 (postintervention) in the experimental and active control groups. The y-axes for SPANE-P and SPANE-N start at 6, which is the minimum possible score for these subscales. MHC-SF EWB: Mental Health Continuum Short Form emotional well-being subscale; MHC-SF PWB: Mental Health Continuum Short Form psychological well-being subscale; SPANE-N: Scale of Positive and Negative Experiences negative affect subscale; SPANE-P: Scale of Positive and Negative Experiences positive affect subscale; VAS: visual analog scale.

A repeated measures ANCOVA with group condition as a between-subjects factor (experimental group: n=23; active control group: n=22) and time (T0 and T1) as a within-subjects factor was performed for all variables with centered MHC-SF EWB, MHC-SF PWB, SPANE-P, SPANE-N, and relaxation VAS baseline scores as covariates, and each of them was used for each analysis. The time×group interactions were examined as they would indicate a significant change due to the intervention. Descriptive data are shown as mean and SD (Table 2) . The repeated measures ANCOVA indicated significantly greater increases in the experimental group in emotional well-being (MHC-SF EWB; *P*<.001; repeated contrast: *P*<.001), psychological well-being (MHC-SF PWB; *P*<.001; repeated contrast: *P*<.001), positive emotions (SPANE-P; *P*<.001; repeated contrast: *P*<.001), and relaxation (relaxation VAS; *P*<.001; repeated contrast: *P*<.001) compared to the control group. Furthermore, the repeated measures ANCOVA revealed a significant decrease in the experimental group in negative emotions (SPANE-N; *P*<.001; repeated contrast: *P*<.001) compared to the control group. See Figure 4 for emotional and psychological well-being, positive and negative emotions, and relaxation scores at T0 and T1 in both groups.

**Table 2.** Comparison between the experimental and active control groups (T0^a^-T1^b^): repeated measures analysis of covariance interaction effects.

Scale	Experimental group, mean (SD)		Active control group, mean (SD)		Time×group, interaction effect		
	T0	T1	T0	T1	*F* (*df*)	*P* value	Partial *η*^2^
MHC-SF EWB^c^	3.05 (1.38)	3.92 (0.85)	3.83 (3.02)	4.18 (1.01)	13.90 (42)	<.001^d^	0.398
MHC-SF PWB^e^	3.31 (1.15)	4.02 (0.70)	4.09 (0.80)	4.31 (0.71)	26.61 (42)	<.001^d^	0.559
SPANE-P^f^	17.87 (4.84)	23.30 (4.38)	23.86 (4.00)	24.64 (4.88)	14.38 (42)	<.001^d^	0.407
SPANE-N^g^	11.91 (6.05)	9.48 (5.12)	11.09 (5.01)	10.00 (3.61)	17.60 (42)	<.001^d^	0.456
Relaxation VAS^h^	7.13 (2.91)	8.65 (2.12)	7.73 (2.52)	8.95 (1.52)	31.22 (42)	<.001^d^	0.598

a T0: preintervention/baseline.

b T1: postintervention.

c MHC-SF EWB: Mental Health Continuum Short Form emotional well-being subscale.

d Significant (*P*<.05).

e MHC-SF PWB: Mental Health Continuum Short Form psychological well-being subscale.

f SPANE-P: Scale of Positive and Negative Experiences positive affect subscale.

g SPANE-N: Scale of Positive and Negative Experiences negative affect subscale.

h VAS: visual analog scale.

### Long-Term Effects: Psychological Changes at the 1-Month Follow-Up

To assess the sustained psychological improvements from T0 to T2, separate within-subjects Student paired *t* tests were conducted for the experimental group (n=17) and active control group (n=15) considering T0 and T2 across all variables. The Student paired *t* test revealed significant differences between T0 and T2 for the experimental group in emotional well-being (MHC-SF EWB; *P=*.046), psychological well-being (MHC-SF PWB; *P*=.03), and positive emotions (SPANE-P; *P*=.005). No significant differences between T0 and T2 were observed for all variables in the active control group (Tables3  4).

**Table 3.** Within-subjects analysis in the experimental group considering T0^a^ and T2^b^.

Scale	Experimental group, mean (SD)		Paired sample *t* test	
	T0	T2	*t* test (*df*)	*P* value
MHC-SF EWB^c^	3.14 (1.27)	3.53 (1.25)	–2.16 (16)	.046^d^
MHC-SF PWB^e^	3.37 (1.05)	3.90 (0.66)	–2.47 (16)	.03^d^
SPANE-P^f^	18.82 (4.06)	22.65 (5.24)	–3.28 (16)	.005^d^
SPANE-N^g^	10.65 (5.27)	11.00 (4.96)	–0.33 (16)	.74
Relaxation VAS^h^	8.29 (1.72)	7.53 (2.15)	1.51 (16)	.15

a T0: preintervention/baseline.

b T2: 1-month follow-up.

c MHC-SF EWB: Mental Health Continuum Short Form emotional well-being subscale.

d Significant (*P*<.05).

e MHC-SF PWB: Mental Health Continuum Short Form psychological well-being subscale.

f SPANE-P: Scale of Positive and Negative Experiences positive affect subscale.

g SPANE-N: Scale of Positive and Negative Experiences negative affect subscale.

h VAS: visual analog scale.

**Table 4.** Within-subjects analysis in the active control group considering T0^a^ and T2^b^.

Scale	Active control group, mean (SD)		Paired sample *t* test	
	T0	T2	*t* test (*df*)	*P* value
MHC-SF EWB^c^	3.87 (0.76)	3.53 (1.11)	1.08 (14)	.30
MHC-SF PWB^d^	4.01 (0.90)	4.06 (0.67)	–0.16 (14)	.87
SPANE-P^e^	23.47 (4.58)	24.07 (4.14)	–0.46 (14)	.65
SPANE-N^f^	11.40 (5.27)	11.13 (4.50)	0.25 (14)	.80
Relaxation VAS^g^	8.00 (2.10)	7.80 (2.33)	0.29 (14)	.77

a T0: preintervention/baseline.

b T2: 1-month follow-up.

c MHC-SF EWB: Mental Health Continuum Short Form emotional well-being subscale.

d MHC-SF PWB: Mental Health Continuum Short Form psychological well-being subscale.

e SPANE-P: Scale of Positive and Negative Experiences positive affect subscale.

f SPANE-N: Scale of Positive and Negative Experiences negative affect subscale.

g VAS: visual analog scale.

### Relaxation Changes Within Sessions

To investigate differences between the groups in each session, delta scores for self-reported relaxation and SpO_2_ were obtained by subtracting the presession scores from the postsession scores. The Student *t* test for independent samples was performed on delta scores. Both relaxation and SpO_2_ increased in all sessions in both groups, and no significant differences were found between groups (Table 5).

**Table 5.** Relaxation and SpO_2_^a^ levels before and after each session.

Variable		Experimental group, mean (SD)	Active control group, mean (SD)	Independent sample *t* test	
				*t* test (*df*)	*P* value
Relaxation 1					
Presession		6.78 (2.58)	7.64 (2.28)	—^b^	—
Postsession		8.83 (1.11)	9.32 (1.04)	—	—
Delta score		2.04 (2.10)	1.68 (1.84)	0.61 (43)	.54
Relaxation 2					
Presession		6.74 (2.16)	6.86 (2.78)	—	—
Postsession		8.84 (1.50)	9.14 (1.78)	—	—
Delta score		1.74 (2.03)	2.27 (2.29)	–0.83 (43)	.41
Relaxation 3					
Presession		7.43 (2.17)	7.32 (2.66)	—	—
Postsession		8.57 (1.41)	9.14 (1.39)	—	—
Delta score		1.13 (1.77)	1.82 (1.97)	–1.24 (43)	.22
Relaxation 4					
Presession		8.21 (1.78)	8.00 (2.03)	—	—
Postsession		9.21 (1.03)	9.47 (1.12)	—	—
Delta score		1.00 (1.33)	1.47 (1.50)	−1.03 (43)	.31
SpO_2_ 1					
Presession		92.04 (3.57)	92.50 (3.49)	—	—
Postsession		94.26 (2.12)	95.55 (1.71)	—	—
Delta score		2.22 (2.71)	3.05 (2.89)	–0.99 (43)	.33
SpO_2_ 2					
Presession		92.26 (3.63)	93.09 (2.2)	—	—
Postsession		94.87 (2.28)	95.73 (2.16)	—	—
Delta score		2.61 (3.14)	2.64 (2.26)	–0.03 (43)	.97
SpO_2_ 3					
Presession		91.39 (3.09)	93.68 (2.57)	—	—
Postsession		93.91 (3.13)	95.41 (2.20)	—	—
Delta score		2.52 (2.31)	1.73 (2.90)	1.02 (43)	.31
SpO_2_ 4					
Presession		93.42 (1.80)	94.21 (2.23)	—	—
Postsession		95.21 (1.47)	95.74 (1.94)	—	—
Delta score		1.79 (1.93)	1.53 (1.74)	0.44 (43)	.66

a SpO_2_: peripheral oxygen saturation.

b Not applicable.

### IVR Acceptance and Sense of Presence

Regarding IVR acceptance, patients reported high levels of behavioral intention (range 1‐7 for each subscale; mean 5.55, SD 1.91). They also reported high levels of performance expectancy (mean 5.21, SD 1.91) and effort expectancy (mean 5.64, SD 1.54) for this intervention. In addition, patients reported very low levels of VR anxiety when experiencing the intervention (mean 1.16, SD 0.64). Furthermore, patients experienced a high sense of presence (range 1‐7; session 1: mean 5.25, SD 1.45; session 2: mean 5.20, SD 1.77), especially during the last 2 sessions (session 3: mean 5.73, SD 1.32; session 4: mean 5.77, SD 1.69).

### Qualitative Analysis of the VR Experiences and the Savoring Exercises

After each session, we asked patients to freely describe how they had felt during the IVR experience, and we categorized their impressions into thematic areas. These thematic areas and the in-depth analysis of the contents of the narrated experiences are provided in Section S3 in Multimedia Appendix 1.

Regarding savoring exercises, they stimulated the memory of several positive experiences related to childhood, adolescence, births of children and grandchildren, marriages, travel, day trips, illnesses, and retirement. Some memories were related to specific elements of virtual scenarios that had been seen before, whereas others were not. A qualitative evaluation of the emotions and positive bodily sensations experienced by the patients during the savoring exercises can be found in Section S4 in Multimedia Appendix 1.

### Perceived Usefulness of the Intervention

Regarding perceived usefulness, in the experimental group, 8 patients rated the intervention as completely useful, 4 rated it as very useful, 8 rated it as quite useful, and 2 rated it as a little useful. In the active control group, 2 patients rated listening to relaxing music as completely useful, 13 rated it as very useful, 5 rated it as quite useful, and 1 rated it as not useful at all. A qualitative analysis of patients’ explicit opinions and suggestions about the intervention was also conducted. We identified 6 main categories from the patients’ comments, 4 of which were common to both groups (Table 6).

**Table 6.** Qualitative analysis of the perceived usefulness of the intervention.

Variable	Experimental group (number of patients)	Active control group (number of patients)
Organization and procedures of the intervention	Sessions and timing were adequate (23) Times between sessions were helpful in reflecting on and appreciating the positive memories that came to mind during the sessions (1) Desire to have more sessions and to view longer IVR^a^ scenarios (3)	Sessions and timing were appropriate (21)
Expectations about the intervention	Initial hesitation to begin the intervention, but later reconsidered (2)	—^b^
Intervention strengths	Opportunity to relive very good past moments (2) Opportunity to learn something new (1) Break the monotony of hospitalization (2) Breathing better as a result of the intervention (4)	Listening to music was very relaxing (9) Appreciation for the sessions, even though they preferred other music genres (3) Disconnect from daily hospitalization routine (1) Breathing better after listening to music (1)
Intervention weaknesses	Preference for more realistic scenarios (2)	—
Future expectations	Desire to follow the intervention again in future hospitalizations (6) Desire to financially support the intervention (1)	Request to receive additional relaxing music to listen to even after the conclusion of the intervention (2) Suggestion of other relaxing music to use for future sessions (1)

a IVR: immersive virtual reality.

b Not applicable.

## Discussion

### Emotional and Psychological Well-Being

People with CRDs often face significant challenges due to their conditions, which go beyond the physical aspect and extend to emotional and psychological impacts. Therefore, it is crucial to develop innovative approaches to promote the well-being of patients with CRDs. Taking into account all these elements, this RCT investigated the effectiveness of an innovative intervention involving IVR-based relaxation integrated with savoring in promoting emotional and psychological well-being, positive emotions, and relaxation, and reducing negative emotions in patients with CRDs. The presence of psychological improvements at the 1-month follow-up compared to the baseline was also investigated.

The findings are very encouraging and partially support our hypothesis. Regarding the primary outcomes, such as the effectiveness of the intervention for increasing emotional and psychological well-being (hypothesis 1), patients reported significant improvements, and these results align with the findings of other studies in which VR scenarios were used to promote emotional and psychological well-being [31] and studies involving patients with chronic diseases [70]. Indeed, the 3 immersive virtual experiences took patients on a first-person exploration of natural surroundings, guided by a soothing narrative voice that helped them focus on their positive bodily and emotional sensations [31]. In this regard, savoring may have contributed to amplifying the positive states experienced during the virtual experiences. This suggests that the intervention may have facilitated patients’ connection with the thoughts and emotions experienced during the virtual scenarios. Furthermore, through the savoring exercises, patients may have had the opportunity to reflect on their own positive memories, savor them, and amplify them deeply.

### Positive and Negative Affect

Regarding the intervention’s effect on positive and negative affect, the second hypothesis (hypothesis 2) was supported. Indeed, the intervention was effective in increasing positive emotions. As highlighted by previous studies, natural environments and nature-related auditory elements included in the VR experiences are effective in promoting positive emotions [29]. They are also successful in creating effects comparable to restorative natural surroundings [30,71] and are particularly suitable for hospitalized patients who cannot move outside the hospital. Furthermore, the savoring exercises may be especially effective for older patients who have several positive memories in which they can immerse themselves [72]. These findings also align with previous savoring interventions targeting older adults, where participants were able to recall and savor positive past events and were able to experience in the present the positive emotions experienced then [40,42,72]. The intervention was also effective in decreasing negative emotions. Previous studies have shown both IVR [73] and savoring to be effective in reducing negative emotions [74,75]. This may be due to the positive emotions experienced both in IVR and during savoring exercises, which may help counterbalance negative emotions related to the pathology and hospitalization, in line with the undoing hypothesis [37].

### Relaxation

Concerning the effectiveness of the intervention in increasing relaxation (hypothesis 3), patients reported significant improvements. This result aligns with the findings of other studies demonstrating the IVR relaxation effect, especially when including natural and peaceful settings and natural auditory elements [20,29]. Indeed, relaxing virtual scenarios have already been used in patients with CRDs, showing positive effects on depression, anxiety, and stress [35]. These findings suggest a potential contribution of the virtual scenarios developed by Become-Hub to hospitalized patients’ reported relaxation.

### Long-Term Effects: Psychological Improvements at the 1-Month Follow-Up

Concerning the long-term psychological improvements from baseline to the 1-month follow-up, the fourth hypothesis (hypothesis 4) was supported, suggesting the positive long-term effects of the intervention on emotional and psychological well-being, and positive emotions. These findings indicate that the intervention may have supported patients in coping with daily life after hospitalization, and savoring may have contributed to this effect. Indeed, the savoring exercises learned during the intervention may have supported patients in developing, maintaining, and amplifying positive feelings, which in turn may have positively influenced their well-being over time. In particular, savoring practice can motivate patients to be engaged in a broader range of thoughts and behaviors, and it allows for the development of long-term resources [39,76,77]. Given the psychological burden associated with CRDs, as widely documented in several literature reviews [78-80], using such a tool in daily life may be a valuable resource in supporting patients even after hospital-based pulmonary rehabilitation.

### Relaxation Within Sessions

Regarding the secondary outcomes, such as the intervention’s effect on self-reported relaxation and SpO_2_ pre- and postsession (hypothesis 5), the absence of differences between the groups can be understood in light of the positive effects of music on relaxation [17], already positively tested in patients with COPD [81,82]. It seems that the effects of the intervention involving IVR-based relaxation integrated with savoring and the use of relaxing music are similar in the short term (postsession). In fact, SpO_2_ levels increased in both groups immediately after the sessions, but without significant differences between the groups. Both music and VR-based relaxation may have induced participants to breathe more deeply, and therefore, these findings suggest that both conditions might activate similar physical mechanisms associated with relaxation, such as increased heart rate variability, respiratory rate, and cortisol levels, which, in turn, may contribute to improved SpO_2_ in the short term (postsession) [83-86]. By contrast, the IVR-based intervention demonstrated greater effectiveness than listening to music over the 2-week intervention, and this could be due to its engaging properties, which allow patients to reflect and amplify their positive experiences, while listening to music has immediate positive effects but is less effective considering a 2-week period.

### IVR Acceptance and Sense of Presence

Regarding IVR acceptance, patients reported a favorable intention to experience the intervention involving IVR-based relaxation integrated with savoring during their future hospitalizations. They also showed a high performance expectancy for this intervention, evaluated it as easy and understandable, and reported very low IVR anxiety. These findings align with previous studies that used IVR or savoring in people of similar age [71,87,88]. Patients experienced a high sense of presence in all 4 sessions, and these results align with previous research [59,89]. Indeed, qualitative analysis revealed that patients experienced mainly positive bodily sensations related to IVR and the sense of being there in the virtual environments. Moreover, the IVR scenarios helped several patients breathe deeper and promoted principally positive affective states, including tranquility, peace, and safety. Many patients felt relaxed and were able to connect specific elements of the scenarios to positive past memories, suggesting that virtual experiences can help people in this process through autobiographical recall. Indeed, when the sense of presence and the personal importance of IVR experiences are considered, they may be experienced in a way that is personally relevant [90], leading to the creation of a connection with the personal experiences.

Qualitative analysis of the savoring exercises also revealed that patients mainly savored positive memories shared with loved ones from the past (eg, their parents or grandparents) and present (eg, their sons, daughters, and grandchildren). During the savoring exercises, patients experienced several positive emotions and positive bodily sensations related to these emotions. In addition, most patients in both the experimental and active control groups perceived the respective sessions as useful. Specifically, patients in the experimental group appreciated the organization and procedures, and some of them reported breathing better as a result of the intervention, while others requested for the intervention again in future hospitalizations. Regarding the active control group, patients found the organization and procedures of the sessions appropriate and found listening to music very relaxing.

### Strengths and Limitations

Taking into account the findings of this study, it is possible to recognize some strengths. One is that, to the best of our knowledge, this RCT is the first to integrate IVR and savoring and to investigate the effectiveness of an intervention involving IVR-based relaxation integrated with savoring in patients with CRDs for increasing well-being in terms of emotional and psychological well-being, positive emotions, and relaxation, and decreasing negative emotions. In addition, the presence of psychological improvements at the 1-month follow-up compared to the baseline was examined, highlighting the importance of creating well-being promotion interventions that can help patients even after hospitalization and assess their long-term effects. As underlined by Riches et al [30], there is a lack of data in the literature regarding the effects of IVR on relaxation in the long term, and assessing follow-up is encouraged. The presence of an active control group that listened to relaxing music was another strength of this study because it is a widely used technique that has proven to be effective in enhancing mental and physical well-being in patients with CRDs [17,82]. Moreover, the inclusion of an active control group strengthened the methodological design of the study, allowing for a more accurate interpretation of the intervention’s effects. Another strength is the employed methodology, which included several approaches, such as quantitative self-report measurements, objective data, such as SpO_2_, and qualitative data, such as patients’ memories and opinions, in order to have as complete an overview as possible of the intervention’s effects. The session organization and timing were also strengths of this study, probably due to the engaging activities and the 2-week duration that was considered appropriate by patients. However, there were some limitations. First, the sample was heterogeneous, as it included patients with different CRDs. Investigating the intervention effects on specific CRDs could provide a deeper understanding. Second, although baseline differences between the groups were statistically controlled using ANCOVAs with centered scores as covariates, the presence of substantial preintervention imbalances should be acknowledged. Third, the results can be considered partially generalizable as they involved a sample of patients with CRDs from Northern Italy. Fourth, the sample size decreased considerably at the 1-month follow-up, reducing the statistical power. Moreover, given the integrated nature of the protocol that combined IVR and savoring, it is not possible to analyze the contributions of the individual components of the protocol (IVR-based relaxation and savoring). Future studies are encouraged to investigate the roles of both separately. In addition, the occurrence of negative side effects (eg, cybersickness) of IVR was not systematically measured after each session, even if the patients’ qualitative responses mainly highlighted positive bodily sensations after watching the scenario. Furthermore, this study did not include specific outcome measures related to perceived dyspnea or fatigue, which are key symptoms in patients with CRDs. Future research should consider incorporating validated measures of these dimensions to better explore the impact of psychological interventions on them. 

### Conclusions

In patients with CRDs, an intervention involving VR-based relaxation integrated with savoring is effective in enhancing emotional and psychological well-being, positive emotions, and relaxation and in reducing negative emotions, and its effectiveness is even higher than that of widely used techniques such as listening to relaxing music [15,17]. The findings and the appreciation for the intervention highlight the potential of IVR integrated with savoring. However, given the integrated nature of the protocol (IVR-based relaxation combined with savoring), it is not possible to disentangle the specific contribution of each component. Therefore, while savoring may play a role in amplifying and extending positive emotional experiences beyond the virtual scenario, potentially helping patients shift their focus and dwell on the positive aspects of their lives, the design of this study does not allow for causal conclusions. Moreover, this intervention underlines the importance of promoting well-being during hospitalization. Indeed, the findings of this study offer important insights for the potential implementation of this intervention within pulmonary rehabilitation programs. These programs, which have traditionally focused on improving physical functioning and managing the physical symptoms of CRDs, are increasingly recognizing the importance of addressing the psychological and emotional aspects of CRDs, given the significant psychological burden experienced by patients with CRDs. The current intervention therefore aligns with these goals, and its brief and structured nature makes it suitable for integration into standard respiratory rehabilitation protocols, offering a scalable and engaging tool to support patients not only during hospitalization but also in the transition to home care and self-management. Patients can use this tool in their daily lives and in future difficult situations to recognize and appreciate their positive moments. Notably, the long-term effects of the intervention (at the 1-month follow-up) on emotional and psychological well-being and positive emotions highlight the great potential of this intervention for maintaining well-being over time. Future studies should further examine the sustainability of these effects with a larger sample size to broaden the understanding of the long-term effects and to explore the intervention’s effects on specific CRDs.

## Supplementary material

10.2196/67395 Multimedia Appendix 1 Integrating virtual reality with savoring for patients with chronic respiratory diseases.

10.2196/67395 Checklist 1 CONSORT-EHEALTH checklist (V 1.6.1).

## Abbreviations

ANCOVA: analysis of covariance
CONSORT: Consolidated Standards of Reporting Trials
COPD: chronic obstructive pulmonary disease
CRD: chronic respiratory disease
GDPR: General Data Protection Regulation
INRCA: Istituto Nazionale di Ricovero e Cura per Anziani
IRCCS: Istituto di Ricovero e Cura a Carattere Scientifico
IVR: immersive virtual reality
MHC-SF: Mental Health Continuum Short Form
MHC-SF EWB: Mental Health Continuum Short Form emotional well-being subscale
MHC-SF PWB: Mental Health Continuum Short Form psychological well-being subscale
MMSE: Mini-Mental State Examination
RCT: randomized controlled trial
SPANE: Scale of Positive and Negative Experiences
SPANE-N: Scale of Positive and Negative Experiences negative affect subscale
SPANE-P: Scale of Positive and Negative Experiences positive affect subscale
SpO_2_: peripheral oxygen saturation
SUS: Slater Usoh Steed Presence Questionnaire
T0: preintervention/baseline
T1: postintervention
T2: 1-month follow-up
UTAUT: Unified Theory of Acceptance and Use of Technology
VAS: visual analog scale
VR: virtual reality
