# Supplementary material for: Integrating Immersive Virtual Reality With Savoring to Promote the Well-Being of Patients With Chronic Respiratory Diseases: Pilot Randomized Controlled Trial
Source: J Med Internet Res. 2025 Sep 23;27:e67395. doi: 10.2196/67395 (PMC12456846; doi:10.2196/67395)
Supplement: Multimedia Appendix 1 [file jmir-v27-e67395-s001.doc]

**Multimedia Appendix 1**

**SECTION S1**

**Music used in the active control group**

**Session 1**

- Debussy *Prelude to the Afternoon of a Faun* (McBride, 1999)
- Bach *Largo from Concerto for Two Violins* (McBride, 1999)

**Session 2**

- *Chinese Bamboo Flute with Nature Sounds* (Hill & Frederick, 2016)

**Session 3**

- Mozart *Symphony #40* (2^nd^ movement) (McBride, 1999)
- Pachebel *Canon in D Major* (Knight & Rickard, 2001)

**Session 4**

- Percy Faith *Theme From a Summer Place, Ebb Tide, Sound of Music* (McBride, 1999)
- Wayne Gratz *Reminiscence, Time Out* (McBride, 1999)
- David Lanz *Cristofori’s Dream* (McBride, 1999)

**SECTION S2**

**Example of a savoring exercise after the VR experience**

**Session 1 – Participants were invited to recall and savor a positive past experience**

Good afternoon, to immerse yourself more in this activity I recommend that you close your eyes, but if you prefer you can also keep them open. Now try to think of what positive emotions and feelings this scenario evoked in you, such as tranquility, peace, calmness, relaxation, serenity, harmony, and wonder. I now ask you to think of a positive past episode or event in which you experienced similar emotions. Now take a moment to focus on the chosen positive experience.

If a positive event has come to your mind, we can continue with the exercise. If you need a few more minutes to recall the experience let the researcher know. Now focus on the state of mind you experienced, imagine what thoughts went through you, recall the expression, posture, breath, sensations you experienced in your body on that occasion--as if it were occurring right now. In this image, notice whether you are observing yourself from the outside or whether you are observing yourself firsthand. Observe whether your point of view is from above, from below, from the side, from the front, from behind. Notice at what distance is the scene you visualize; it might be near or far. It might be colored or black and white, still or moving, in focus or blurred, bright or dark. Observe whether details are present or whether the scene is sketchy. There might be sounds or noises, foreground or background. Pay attention now to your breathing, how its rhythm has changed, whether it is slower or faster. Notice where the most intense sensation is in the body (in the shoulders, legs, torso, arms, hands), whether it is diffuse or in specific places, whether it is still or moving.

Now try to slowly reactivate your body starting with the movement of your hands and feet, and if your eyes are closed, gently open them again. Now that you have finished the activity, try to notice the sensations that the exercise has aroused in you and the emotions you have felt and are feeling.

**SECTION S3**

**Qualitative analysis of the virtual reality experiences**

After each session, we asked patients to freely describe how they had felt during the VR experience, and we categorized their impressions into some thematic areas (Table S1).

**Table S1.** Qualitative evaluation of the VR experiences: principal themes and experiences (number of patients).

| **Themes** | **Experiences (number of patients)** |
| --- | --- |
| **Bodily sensations** | Floating pleasantly within the virtual scenario (3)  Being on a chairlift and perceiving peace and tranquillity all around (2)  Sensation of flying (1)  Little nausea (2)  A slight feeling of lurching while viewing the virtual scenario (1) |
| **Immersion** | Feeling like touching cherry blossoms in the secret garden (1)  Desire to lay on the lawn of the secret garden (1)  Desire to lay on the hammock in the beach at sunset (2)  Feeling the warmth of the sun and the sea breeze on the skin during the beach at sunset (1) |
| **Deep breathing** | Feeling of breathing more deeply and easily (5)  A participant rethought about the beach at the sunset during the pulmonary exercise training helped him walk 4 more laps than his daily goal (1) |
| **Affective states** | Experiencing tranquillity during the secret garden and the prairie waterfall (1)  Feeling peace during the beach at sunset (1)  Feeling safety due to the presence of the large tree and village in the distance during the waterfall in the prairie (1)  Slight fear of the clear water of the pond due to the inability to swim during the waterfall in the prairie (1) |
| **Relaxation** | Experiencing relaxation (9)  Feeling a relaxing sensation in the ankles (1)  Experiencing feelings of newness and well-being (1)  The VR experience was slower than the busy life (1) |

Starting from the patients’ stories, it can be seen that specific elements of the scenarios, particularly natural elements, elicited memories of positive past events containing similar natural elements.

For example, regarding the “secret garden” VR scenario, the waterfall reminded one patient of waterfalls he had seen in Mexico and Germany. In another patient, the lake recalled a Nile cruise she took a few years earlier, while a patient, seeing the cherry trees, thought of an orchard she saw as a teenager. Some artificial elements encountered during the VR experience were also linked to positive past and future experiences. For example, the garden fence reminded one patient of the fence he wants to build for her daughter's house, while one patient connected the view of the little bridge over the lake to an experience in an oriental museum she had in Scotland.

In the “waterfall in the prairie” VR scenario, the sight of tulips reminded one patient of a desire to plan a trip with family members to go see them. The lake and crystal-clear water, on the other hand, reminded one patient of Monet's paintings he saw a few years earlier, while another patient remembered of a vacation spent on a Maldives atoll with his wife, where he was able to see the beautiful colours of the sea and landscape, and when he cried for the beauty of that moment. Furthermore, the meadow in the virtual scenario also prompted many memories related to trips and vacations taken in the mountains as children or adults.

In “the beach at sunset” VR scenario, the sea reminded many patients of vacations where they were able to see a seascape with similar colours, including trips to Santo Domingo and Sri Lanka. Furthermore, for one patient the very light colour of the sand reminded her of snow. Finally, many patients connected the starry sky of the scenario to situations in which they had seen a similar sky: one patient, for example, recalled a vacation when she was young with her friends where one night they saw a beautiful starry sky. Regarding perceived realism of the virtual scenarios, 3 patients considered unrealistic some elements of the secret garden (e.g., the carps and the grass), of the waterfall in the prairie (e.g., the river, the clouds, the wind, and the grass), and two of them of the beach at sunset (e.g., the sun and the starfish).

**SECTION S4**

**Qualitative analysis of the savoring exercises and emotions experienced during each session**

In the first savoring exercise, patients were asked to recall and savor a positive memory. For example, one patient narrated about his grandchild’s birth, and another patient thought about the successful outcome of his mother's cancer operation. There were also many narratives pertaining to travel as that of a patient who linked the cherry blossom trees of the virtual experience (the secret garden) to his trip to Kyoto with his daughter during the cherry blossom month.

In the second savoring exercise, patients were asked to savor a positive past event shared with a loved one. Many patients connected the scenario (the waterfall in the prairie) to day trips, such as a patient who recalled in detail a picnic in the mountains with her parents and grandmother when she was 7 years old. After recalling this experience, the patient was moved because she felt all the love from her parents and was amazed that she was able to remember an event from 60 years earlier that she had never thought back to.

In the third savoring exercise, patients were invited to mentally create and savor a personal nice place. Most of the patients linked their memories to the theme of the VR scenario (the beach at sunset) by telling about the experience at the sea and/or in front of starry skies. Moreover, a patient connected the beach sand of the scenario to the desert sand he saw on a trip abroad and stated, "There was a Bedouin tent with rugs and pillows where I could rest and was offered tea. There was also a salt lake with a white bottom and crystal-clear water, fish, dromedaries around the lake and elegant white cranes. I heard the sound of the wind, the verses of the dromedaries, the hubbub of two children with a horse under a tree, and the silence of the desert.”

In the fourth exercise, patients were guided through the exercises completed in the previous sessions to consolidate them. For instance, a patient related the various elements of the scenario she chose to review (the secret garden) to specific moments in her life and reported, "The butterflies reminded me of when I had the net bought as a child to catch butterflies in the garden. The fish reminded me of when I used to go to the amusement park and while playing I won a goldfish. Water lilies reminded me of a lily pad I drew in eighth grade. The stones reminded me of the less good things in his life until retirement, while the green lawn reminded me of the good things in my life that were more than the bad ones. The bamboo door reminded me of the longed-for retirement that I achieved."

In this framework, the savoring exercises have stimulated the memory of several positive experiences related to childhood, adolescence, births of children and grandchildren, marriages, travel, day-trips, illnesses, and retirement. Some memories were related to specific elements of virtual scenarios they had seen before, whereas others were not. Many patients also freely reported the emotions and positive bodily sensations felt during the savoring exercises (Table S2).

**Table S2.** Qualitative evaluation of the savoring exercises: emotions and positive bodily sensations (number of patients).

| **Themes** |  | **Experiences (number of patients)** |
| --- | --- | --- |
| **Emotions** | Positive | Joy (6)  Awe (4)  Love (3)  Commotion and crying (3)  Peace (2)  Tranquillity and calmness (2)  Harmony (1)  Gratitude (1) |
|  | Negative | Sadness (2)  Melancholy (1) |
| **Positive bodily sensations** |  | An inner tsunami (1)  A volcano inside (1)  A positive stomach emptiness of relaxation (1)  Goosebumps on arms (1)  Fluidity (1)  A floating sensation (1) |

Regarding the emotional experience after the savoring exercises, many patients specified the positive emotions they felt such as joy, awe, love, peace, and gratitude. Some patients also reported the positive bodily sensations associated with the positive emotions felt. Few patients experienced negative emotions such as sadness thinking back to loved ones who are gone or melancholy thinking back to the carefree days of childhood. To learn more about the complexity of the emotional experience reported by the patients, see section S4.

After each session, patients were asked to rate the intensity of 5 emotions felt during the session (love, awe, enjoyment, gratitude, and hope) on a 10-point VAS. Patients in the experimental group experienced high levels of love especially during the second session. Furthermore, they reported high levels of awe, particularly in the first session, gratitude, especially in the fourth session, and hope, particularly in the fourth session. Enjoyment was the emotion patients felt the least (Figure S1).


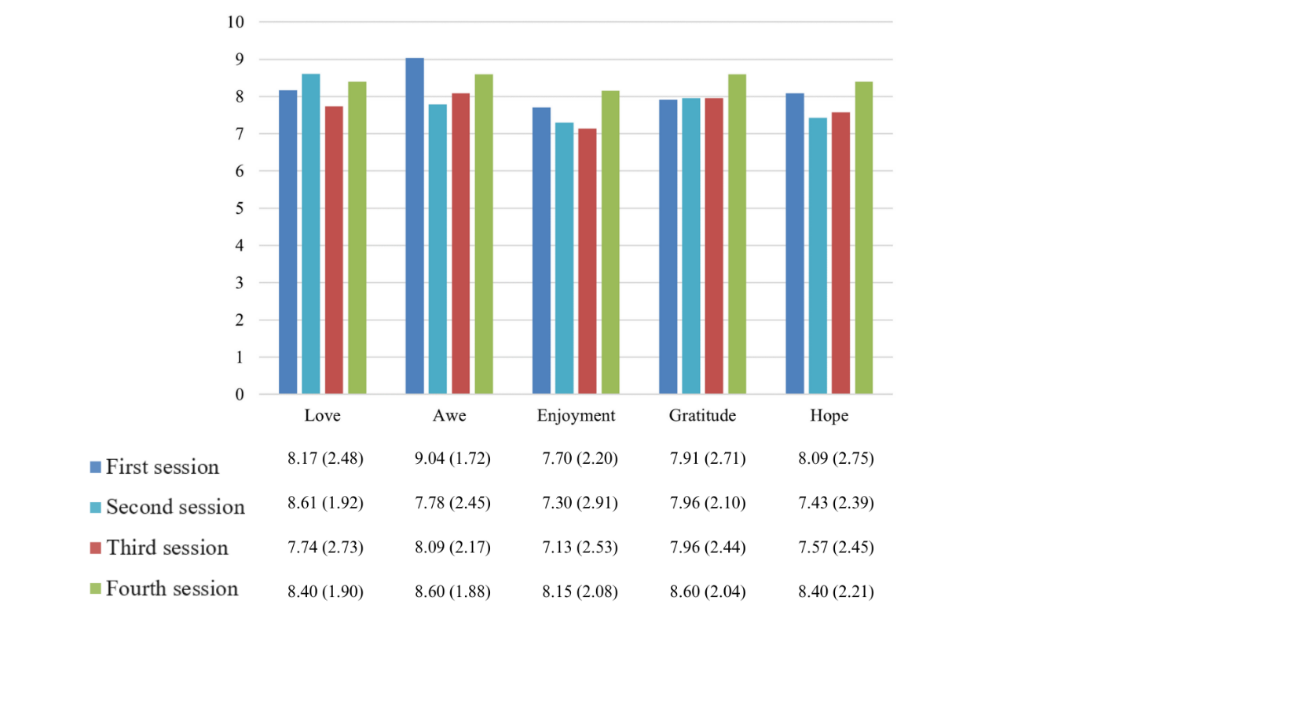
**Fig. S1.** Emotions experienced after each session in the experimental group, mean (M) and standard deviation (SD).
